# Cinnamaldehyde, a Promising Natural Preservative Against *Aspergillus flavus*

**DOI:** 10.3389/fmicb.2019.02895

**Published:** 2019-12-18

**Authors:** Su Qu, Kunlong Yang, Lei Chen, Man Liu, Qingru Geng, Xiaona He, Yongxin Li, Yongguo Liu, Jun Tian

**Affiliations:** ^1^College of Life Science, Jiangsu Normal University, Xuzhou, China; ^2^Beijing Advanced Innovation Center for Food Nutrition and Human Health, Beijing Technology and Business University, Beijing, China

**Keywords:** cinnamaldehyde, *Aspergillus flavus*, antifungal, apoptosis, food preservation

## Abstract

The problem of food spoilage due to *Aspergillus flavus* (*A. flavus*) needs to be resolved. In this study, we found that the minimum inhibitory concentration of cinnamaldehyde (CA) that inhibited *A. flavus* was 0.065 mg/ml and that corn can be prevented from spoiling at a concentration of 0.13 mg/cm^3^. In addition to inhibiting spore germination, mycelial growth, and biomass production, CA can also reduce ergosterol synthesis and can cause cytomembrane damage. Our intention was to elucidate the antifungal mechanism of CA. Flow cytometry, fluorescence microscopy, and western blot were used to reveal that different concentrations of CA can cause a series of apoptotic events in *A. flavus*, including elevated Ca^2+^ and reactive oxygen species, decrease in mitochondrial membrane potential (Δψ*_*m*_*), the release of cytochrome c, the activation of metacaspase, phosphatidylserine (PS) externalization, and DNA damage. Moreover, CA significantly increased the expression levels of apoptosis-related genes (*Mst3*, *Stm1*, *AMID*, *Yca1*, *DAP3*, and *HtrA2*). In summary, our results indicate that CA is a promising antifungal agent for use in food preservation.

## Introduction

*Aspergillus flavus* (*A. flavus*) is one of the most common species among the filamentous fungi. In addition, *A. flavus* is reported to be the second largest cause of aspergillosis infection in humans (Varga et al., 2011). The notorious *A. flavus* metabolite aflatoxin B_1_, which has been recognized by the World Health Organization (WHO) as a primary carcinogen, is absorbed by humans and animals through contaminated agricultural crops and animal feed, such as maize, peanuts, nuts, cottonseed, and edible oil (Yang et al., 2015). Therefore, controlling the food spoilage mediated by *A. flavus* at its source is critical to limiting the health hazards of aflatoxin and to preventing substantial economic losses. Nevertheless, traditional antifungal drugs have continuously posed problems, which include the increasingly serious problem of drug resistance, the toxicity of the chemical antifungal compounds, drug interactions, and the high costs. Research into new antifungal agents needs to be carried out urgently because of the drug resistance and the toxicity of the compounds currently available (Sarkar et al., 2014; Qu et al., 2019). Consequently, increasing numbers of scientists are exploring novel natural products from medicinal plants such as Geraniol and Citral in an attempt to solve the question of fungal drug resistance and with consideration for the natural low toxicity and high antifungal activity of these products (Atanasov et al., 2015; Tang et al., 2018).

Apoptosis is a form of cell death that plays a vital role in the normal development and maturation cycle. In routine physiological processes, the homeostatic balance between cell proliferation and cell death is critical (Fuchs and Steller, 2011). Some scholars have suggested that phenolics can damage mitochondrial function through targeting antioxidative signal transduction and thereby inhibit pathogenic *Aspergillus* (Kim et al., 2004, 2006). Our own previous studies have indicated that apoptosis-promoting compounds are a promising direction in the exploration for a novel antimicrobial drug, and many antifungal agents have been investigated through the apoptotic pathway, such as amphotericin B and anacardic acid (Tian et al., 2017; Qu et al., 2019). In addition, our recent research has shown that Nerol possesses an anti-*A. flavus* ability through apoptosis. Other researchers have indicated that cinnamaldehyde (CA) can decrease the expression of the aflatoxin biosynthetic gene and inhibit the biosynthesis of aflatoxin B1 (Liang et al., 2015; Tian et al., 2018).

Cinnamaldehyde is an α, β-unsaturated aldehyde, abundant in cinnamon and widely used as a food additive in products such as drinks, candies, ice cream, chewing gum, and condiments (Cabello et al., 2009). Furthermore, CA is a traditional Chinese medicine used for gastritis, indigestion, blood circulation disorders, and inflammation (Liao et al., 2012; Chen et al., 2019). CA has been reported to inhibit *Geotrichum citri-aurantii* in citrus fruits and *Phytophthora capsici* in peppers, both of which result in food decay (Hu et al., 2013; OuYang et al., 2019). CA is well-tolerated in humans and animals and is considered a safe natural active ingredient. The FDA and the council of Europe have accepted this concept and recommend daily intake of 1.25 mg/kg (Zhu et al., 2017). Furthermore, CA has been reported to remove natural or chemical toxicities such as ochratoxin A and to protect human health (Dorri et al., 2018; Wang et al., 2018a). The antioxidant activity and the anti-cerebral thrombosis ability of CA have been proven in mice (Zhao et al., 2015; Buglak et al., 2018). Some reports have indicated that CA can initiate the production of reactive oxygen species (ROS) and damage the mitochondrial membranes of *Penicillium expansum* (Wang et al., 2018b, c). The use of CA as a preservative in food storage and transportation is widely recognized to be beneficial. In a recent publication, CA is reported to inhibit *A. flavus* at lower concentrations, and CA has also been recognized as able to induce apoptosis in cancer cells (Roth-Walter et al., 2014; Li et al., 2015). Another report indicates that CA mediates *A. flavus* oxidative stress, but it only detected changes in antioxidant enzyme activity, and the follow-on mechanism of ROS in *A. flavus* is not clear (Sun et al., 2016). Nevertheless, the mechanism by which CA inhibits *A. flavus* is considered worth exploring. Therefore, this research investigated the apoptotic effects of CA in *A. flavus*, such as intracellular ROS, calcium concentration, mitochondrial membrane potential, cytochrome c, phosphatidylserine, metacaspase, and DNA damage.

## Materials and Methods

### Materials and Strain

Cinnamaldehyde (CAS registry no. 104-55-2) was purchased from Shanghai Macklin Biochemical, Co., Ltd. (Shanghai, China) and prepared as a stock solution in 0.1% (*v/v*) Tween-80. The *A.* flavus (NRRL 3357) used in this research was purchased from the China General Microbiological Culture Collection Center (CGMCC). It was cultured in potato dextrose agar (PDA: 200 g peeled potato, 20 g dextrose, 15 g agar powder, and 1000 ml distilled water) for 4 days at 28°C and stored at 4°C.

### Antifungal Susceptibility Testing

Broth microdilution methods are commonly used for *in vitro* antifungal assays (Tian et al., 2017). For our experiments, 80 μl of different concentrations of CA, 100 μl of potato dextrose broth (PDB), and 20 μl 5 × 10^6^ spores/ml *A. flavus* were added to each of 10 wells. The 11th well was used as a blank control without the CA, and the 12th well was used as a negative control, without the fungal suspension. After incubation at 28°C for 48 h, the minimal drug concentration that inhibited the growth of the *A. flavus* was described as its minimum inhibitory concentration (MIC).

### Effect of CA on *A. flavus* Pathogenicity in Corn

Fungal infection in corn was investigated using a method described previously with minor modifications (Yang et al., 2018). After the tip of each corn kernel was scratched with a knife, it was immersed in 5% sodium hypochlorite and then placed in a shaker for 10 min. The corn was washed twice with sterile water, twice with 70% ethanol, and again twice with sterile water. It was finally shaken with *A. flavus* for 30 min. Six corn kernels were placed in each Petri dish, and then sterile water and various concentrations of CA were added, and sterile filter paper was put it on the bottom of the plate. For the control, the corn was not treated with CA, and neither was it co-incubated with *A. flavus*. All samples were incubated for 5 days at 28°C after sealing.

### Fungal Culture Conditions

Fungal cells were suspended in phosphate buffered saline (PBS) and adjusted to 5 × 10^6^ spores/ml with a hemocytometer. The PDB and different concentrations of CA (0, 0.033, 0.065, 0.13, 0.26, and 0.52 mg/ml) were mixed and added to the *A. flavus* spore suspension; they were then cultured in a shaker at 28°C for 6 h. At least 200 spores were observed in each treatment group, confirming the spore germination. Nine mm agar disks were prepared on an *A. flavus* plate with a puncher and placed in the center of a PDA medium with the CA at 28°C. The colony diameters were measured after 3 days to measure mycelial growth. The cells were cultured at a constant temperature in a shaker at 28°C for 72 h, and then the hyphae were collected. The hyphae were treated in an oven at 60°C for 24 h and then weighed to determine their biomass.

### Effect of CA on Biofilm of *A. flavus*

*A. flavus* cells were treated with various concentrations of CA for 12 h at 28°C. The morphological changes in the *A. flavus* were observed and analyzed by the forward-scattered light (FSC) and the side-scattered light (SSC) channels of flow cytometry (BD Biosciences, San Jose, CA, United States). As in the previous method, the content of ergosterol in the *A. flavus* cell membrane was analyzed by spectral scanning (Tian et al., 2012). Membrane integrity was determined by monitoring the uptake of fluorescent nuclear staining propidium iodide (PI)—a DNA-stained fluorescent probe. Cells were incubated with 5 μg/ml PI for 30 min at room temperature in the dark and detected by an Accuri C6 flow cytometer (BD Biosciences, San Jose, CA, United States). *A. flavus* cells were centrifuged, and the supernatant was taken out. After dilution, the OD_260 nm_ value was measured for soluble content release by using an ultraviolet-visible spectrophotometer (Thermo Fisher Scientific, Waltham, MA, United States).

### ROS, Δψ*_*m*_*, and Ca^2+^ Measurement

The *A. flavus* cells treated with CA were analyzed by flow cytometry with DCFH-DA (Sigma-Aldrich, St. Louis, MO, United States) to detect the production and accumulation of ROS. JC-1 (Sigma-Aldrich, St. Louis, MO, United States) staining was used to measure Δψ*_*m*_*. Fluo-3/AM (Sigma-Aldrich, St. Louis, MO, United States) and Rhod-2/AM (Sigma-Aldrich, St. Louis, MO, United States) are commonly used to detect cytoplasmic and mitochondrial Ca^2+^ levels (Yun and Lee, 2016). The cells treated with different concentrations of CA (0, 0.033, 0.065, 0.13, 0.26, and 0.52 mg/ml) at 28°C for 12 h were harvested by centrifugation at 5000 × *g* for 5 min, washed twice, and then resuspended in PBS. Cells were then incubated with 10 μM DCFH-DA, 10 μg/ml JC-1, Fluo-3/AM and Rhod-2/AM at 28°C for 30 min in the dark. Finally, the cells were washed in PBS and then analyzed using an Accuri C6 flow cytometer (BD Biosciences, San Jose, CA, United States).

### Analysis of Cytochrome c Release

The *A. flavus* cells were treated with various concentrations of CA for 12 h at 28°C for the detection of cytochrome c. The cells were then harvested, and mitochondrial and cytosolic fractions were prepared with an ultrasonic cell disruptor. Mitochondrial fractions were collected by using a filamentous fungus mitochondrial protein extraction kit (BestiBio, Shanghai, China), and cytoplasmic proteins were collected with a filamentous fungal cytoplasmic protein extraction kit (BestiBio, Shanghai, China). The protein concentration was tested using a microplate reader and a bicinchoninic acid (BCA) Protein Assay Kit (Solarbio, Beijing, China). Sixty micrograms of total cellular proteins were separated by 15% sodium dodecyl sulfate (SDS) polyacrylamide gel electrophoresis and transferred to the polyvinylidene difluoride (PVDF) membrane (Merck Millipore, Billerica, MA, United States). The PVDF membrane was blocked with 5% non-fat milk (m/v) for 1 h and then washed with 0.1% Tween-20 in Tris saline buffer. It was then incubated with rabbit anti cytochrome c (Proteinsimple, Silicon Valley, CA, United States) and mouse anti-GAPDH (Bioss, Beijing, China) for 12 h at 4°C. The membrane was investigated with western blot chemiluminescence reagents (GE Healthcare, Little Chalfont, United Kingdom), and the reactive density was measured using ImageJ software 1.48 V.

### Detection of Metacaspase Activity

Activated metacaspases in *A. flavus* cells were measured with the CaspACE FITC-VAD-FMK *in situ* marker (Promega, Madison, WI, United States). *A. flavus* cells were treated with various concentrations of CA for 12 h at 28°C. The cells were then harvested by centrifugation at 5000 × *g* for 5 min, washed twice, and then resuspended in PBS. The cells were stained with 10 μM of CaspACE FITC-VAD-FMK for 30 min at room temperature in the dark. Finally, the samples were analyzed by using an Accuri C6 flow cytometer (BD Biosciences, San Jose, CA, United States).

### Detection of PS Externalization

PS externalization was detected by fluorescence microscopy using Annexin V-FITC and PI. The method used to prepare the protoplasts has been described in a previous study (Tian et al., 2018). Subsequently, the protoplasts were treated with CA for 12 h at 28°C. Next, the CA-treated protoplasts were stained with 5 μl/ml of PI and FITC-labeled Annexin V, and then analyzed using the Annexin V-FITC apoptosis detection kit (BD Biosciences, San Jose, CA, United States). Finally, the test protoplasts were analyzed by using an Accuri C6 flow cytometer (BD Biosciences, San Jose, CA, United States).

### Analysis of DNA and Nuclear Damage

The TUNEL assay and DAPI staining were used to confirm the diagnostic markers of yeast apoptosis, including DNA and nuclear fragmentation. *A. flavus* cells were treated with various concentrations of CA for 12 h at 28°C. For the DAPI staining, the CA-treated cells were permeabilized and fixed with 70% absolute ethanol at 4°C for 30 min and then treated with 5 μg/ml DAPI (Sigma-Aldrich, St. Louis, MO, United States) for 10 min in the dark. Cells were then harvested and examined under fluorescence microscopy (Leica, Wetzlar, Germany). For the TUNEL assay, 20 μl of the *A. flavus* suspension was added to the adhering slide, and then 100 μl of 4% paraformaldehyde was added, dropwise. Subsequently, 100 μl of 0.2% Triton X-100 and 50 μl of the reaction system were added according to the instructions with the TUNEL kit (Solarbio, Beijing, China). The DNA breaks were observed under fluorescence microscopy (Leica, Wetzlar, Germany).

### Quantitative Real-Time PCR

Total RNA was extracted using the TRI reagent method. RNA was extracted from the mycelium after growth in a liquid culture to ensure that a high yield was obtained with purity of RNA (Canciani et al., 2017). The *A. flavus* mycelia were treated with 0, 0.065, 0.13, and 0.26 mg/ml CA for 12 h, then collected, washed, and resuspended in sterile PBS. The collected mycelia were then fragmented with liquid nitrogen and transferred to a TRI reagent solution. We used a Micro UV spectrophotometer (Thermo Fisher Scientific, Waltham, MA, United States) to test at 260 and 280 nm, and then the RNA was sequentially reverse transcribed to the first strand of cDNA by using the RevertAid First Strand cDNA Synthesis Kit and following the manufacturer’s instructions (Thermo Fisher Scientific, Waltham, MA, United States). The obtained cDNA was used in the analysis of real-time PCR (RT-PCR). The primers used in this study are presented in Table 1. SYBR Green was used (Takara, Tokyo, Japan), and the procedures for Q-PCR were performed on AB (Applied Biosystems, Thermo Fisher Scientific, Waltham, MA, United States) and consisted of denaturation at 95°C for 30 s and 40 cycles of 95°C for 5 s and 60°C for 40 s. The expression level of the target genes relative to the reference was determined by using 2^–Δ^ ^Δ^ ^Ct^ (Jahanshiri et al., 2012).

**TABLE 1:** Names and nucleotide sequences of primers used for RT-PCR in this study.

**Primer name**	**Sequence (5′–3′)**
Mst3-Forward	TGTGCATCTGGCTTGGCTTA
Mst3-Reverse	ATGGTGGGTGCTTTGACTGT
Stm1-Forward	ATTGCCTGCAACAGCGAATC
Stm1-Reverse	CTTCCTGAGTTGCGCCCTAT
AMID-Forward	TTGCGAACCGAGGCTGAATA
AMID-Reverse	ATTGGGACTCGCAGGTTCTC
Yca1-Forward	GTATTCTTGGGGAGCGCCTT
Yca1-Reverse	CTGCGCAATAGCCTACCAGA
DAP3-Forward	GGAAGACTAGAAGGAGACGCA
DAP3-Reverse	TGGTGTCAGAGGGTCAGGAA
HtrA2-Forward	GGCATGAAGCTGATTGCGTT
HtrA2-Reverse	ATGCCGTCCTTGTGTTTGGA
β-Tubulin-Forward	GCTGGAGCGTATGAACGTCT
β-Tubulin-Reverse	GGCACGAGGGACATACTTGT

### Statistical Analysis

All experiments were performed in triplicate (*n* = 3). One-way ANOVAs and Tukey tests were used and data were assessed with GraphPad Prism software, version 8.0.0. The *p*-values were considered significant at <0.05, <0.01, and <0.001.

## Results

### CA Reduced Fungal Viability of *A. flavu*s

**FIGURE 1:**
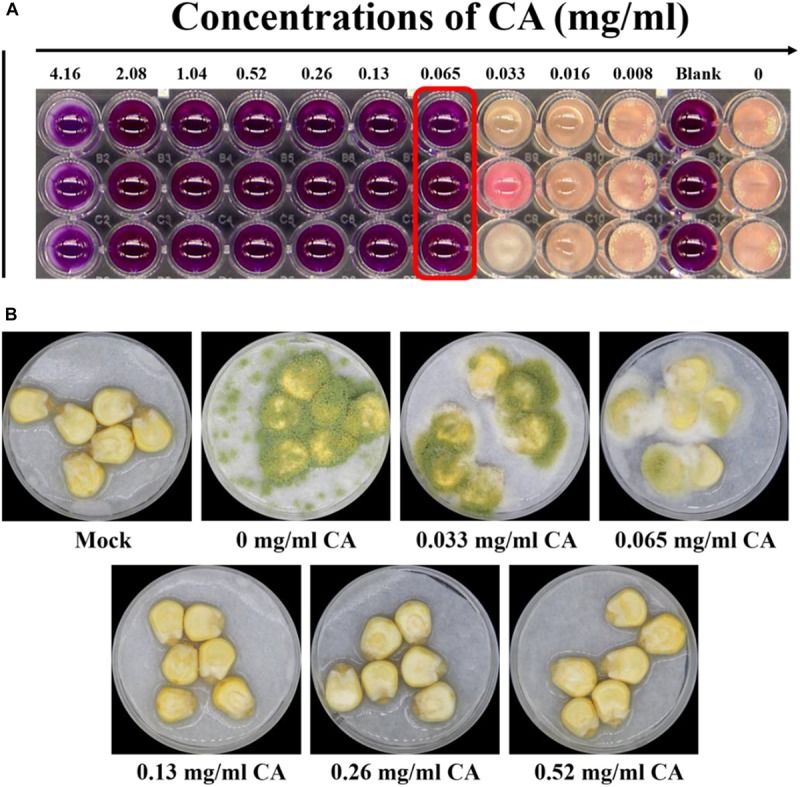
Effects of CA on *Aspergillus flavus* viability and fungal virulence. **(A)** The MIC for *A. flavus* treated with CA was detected by the microdilution method, and the endpoint was observed by using resazurin. **(B)** The antifungal effect of CA on corn: 5 × 10^6^ spores/ml suspension of spore in 0.01% Tween 20 was inoculated into corn, which was treated with CA volatilization, and the Petri dish was kept in a moist incubator at 28°C with 12 h cycles of light/dark for 5 days. The mock control was treated with sterile water, and spore were not inoculated into the corn.

The spoilage by *A. flavus* of seed crops and foodstuffs, together with the contamination by aflatoxins produced by this fungus, have caused significant concern to farmers and the food industry. To resolve the problem of food spoilage by *A. flavus*, CA, an α, β-unsaturated aldehyde that is widely used in food additives, was introduced to treat this fungus. We found that *A. flavus* treated with CA showed MIC at 0.065 mg/ml when the viability of the CA treatment was assessed after 5 days according to visual observation (Figure 1A).

We then examined the ability of *A. flavus* to invade maize kernels treated with CA. As shown in Figure 1B, the maize kernels in the control were unspoiled by fungus. In the treatment samples, maize kernels with little CA treatment were seriously invaded by the *A. flavus*, which produced a large amount of green spore. The spoilage of the maize kernels by *A. flavus* was significantly inhibited by treatment with greater concentrations of CA (0.13, 0.26, and 0.52 mg/cm^3^). *A. flavus* failed to colonize the maize kernels after they were treated with a concentration of 0.13 mg/cm^3^ CA (Figure 1B). At concentrations of 0.26 and 0.52 mg/cm^3^, CA prevented all fungal spoilage. Our results indicated that CA is a promising agent for preventing the infection of seed crops by *A. flavus*.

### CA Inhibited the Sporulation and Fungal Development of *A. flavus*

**FIGURE 2:**
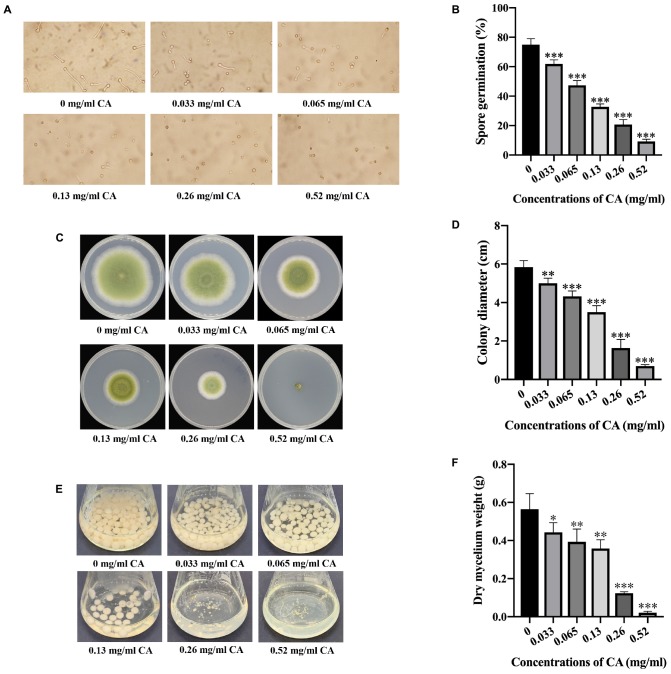
Effects of CA on spore germination and fungal development in *A. flavus*. **(A)** Observation by microscope of spore germination of *A. flavus* treated with different concentrations of CA. **(B)** Statistical analysis of spore germination percentages. **(C)** Mycelial growth of *A. flavus* under various concentrations of CA. **(D)** Statistical analysis of colony diameters. **(E)** The biomass production of *A. flavus* under different concentrations of CA. **(F)** Statistical analysis of dry mycelium weight. In all statistical analysis, ^∗^*p* < 0.05, ^∗∗^*p* < 0.01, and ^∗∗∗^*p* < 0.001 when compared with the 0 mg/ml CA.

To better understand how it is that CA restricts the growth of *A. flavus* in seed crops, the effects of CA on the sporulation and development of *A. flavus* were assayed. As shown in Figures 2A,B, the spore germination of this fungus was significantly inhibited by CA when the concentration was 0.033 mg/ml or more. The results also show that CA had a positive inhibitory effect on the spore germination of *A. flavus*, indicating that the effect of CA on inhibiting sporulation is dose-dependent. In the assay exploring the effect of CA on *A. flavus* development, we found that increasing concentrations of CA significantly inhibited the growth of *A. flavus* (Figures 2C,D). Direct contact with CA significantly inhibited the growth of *A. flavus* hyphae, and this inhibition was positively correlated with the treatment concentration, such that, when the concentration was at 0.52 mg/ml, the mycelial growth of *A. flavus* was completely inhibited. The effect of CA on the biomass of *A. flavus* was also investigated, and this showed that, after treatment with different concentrations of CA, the biomass of *A. flavus* was significantly decreased (Figures 2E,F). The decrease in biomass was positively correlated with the concentration of CA. These results demonstrate that CA has the potential to significantly inhibit the sporulation and fungal development of *A. flavus*.

### CA Destroyed the Biofilm of *A. flavus*

**FIGURE 3:**
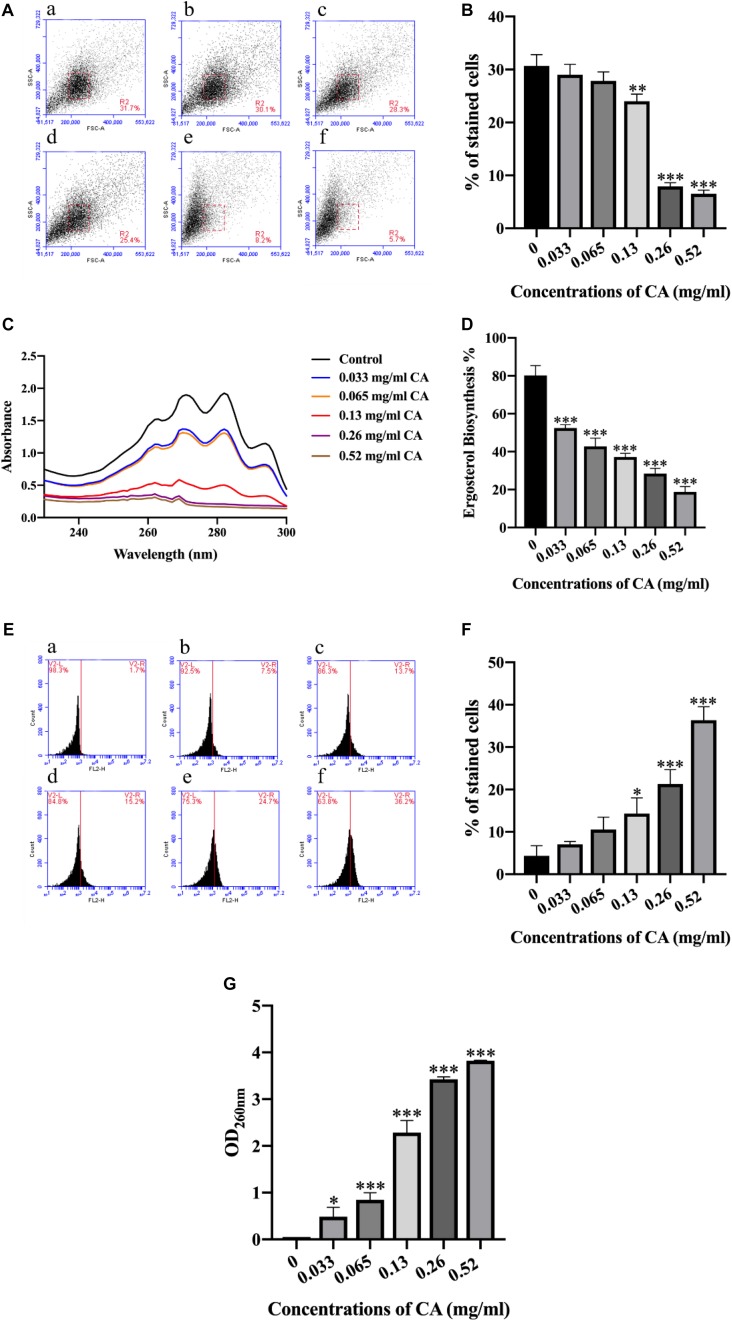
CA destroyed the biofilm of *A. flavus*. **(A)** CA destruction of *A. flavus* cells’ morphology detected by flow cytometry and a histogram analysis of the destruction of cell properties. **(a)** Fluorescence of cells without CA treatment; **(b–f)** *A. flavus* cells exposed to 0.033, 0.065, 0.13, 0.26, and 0.52 mg/ml CA. **(B)** Statistical analysis of percentage of stained cells. **(C,D)** CA inhibited the synthesis of ergosterol, which has been considered a classical antifungal target in *A. flavus* cell membranes. **(E)** PI staining was used to detect the biofilm damage level after being treated with CA and a statistical analysis of the damage to cells. (**a**) Fluorescence of cells without CA treatment; **(b–f)** *A. flavus* cells as treated with 0.033, 0.065, 0.13, 0.26, and 0.52 mg/ml CA. **(F)** Statistical analysis of percentage of stained cells. **(G)** The analysis of *A. flavus* cellular content after being treated with various concentrations of CA. In all statistical analyses ^∗^*p* < 0.05, ^∗∗^*p* < 0.01, and ^∗∗∗^*p* < 0.001 when compared with the 0 mg/ml CA.

As the sporulation of *A. flavus* was dramatically inhibited by CA, we wondered whether the integrity of the cell membrane was impaired by the CA treatment. We then examined the cell morphology of *A. flavus* by using flow cytometry. The FSC (X-axis) indicated the size of the cells, and the SSC (Y-axis) indicated the granularity of the cells. As shown in Figures 3A,B, the morphological characteristics of *A. flavus* cells were changed following treatment with CA, and the extent of the cell changes differed according to the different concentrations of CA. The results showed that CA significantly changed the cell morphology of *A. flavus*.

Ergosterol is the principal sterol in filamentous fungi and it is required for fungal cell membrane growth and normal function (de Lira Mota et al., 2012). We detected the synthesis of ergosterol in *A. flavus*, and the results showed that different concentrations of CA could inhibit the synthesis of ergosterol in the *A. flavus* cell membrane (Figures 3C,D). Compared with the control group, the ergosterol content of *A. flavus* cells decreased after treatment with different concentrations of CA, which indicated that CA inhibition of the synthesis of ergosterol in the *A. flavus* cell membrane was dose-dependent. The cell membrane is an important organelle in cells and plays a key role in material transport and signal transmission. This experiment used PI—a fluorescent dye that can stain nucleic acids—to detect *A. flavus* cell membrane damage after CA treatment. The *A. flavus* cells showed a more pronounced fluorescence as the concentration of CA increased. The optical density (OD) value for detecting the release of the content of *A. flavus* was recorded at a wavelength of 260 nm by an ultraviolet spectrophotometer. As the concentration of CA increased, the OD value obtained from the corresponding experimental group also increased (Figure 3G). The results show that, as the concentration of CA increased, the release of contents also increased. The release of contents after treatment with CA indicates that the cell membrane was destroyed.

### The Accumulation of Intracellular ROS Increased With CA

**FIGURE 4:**
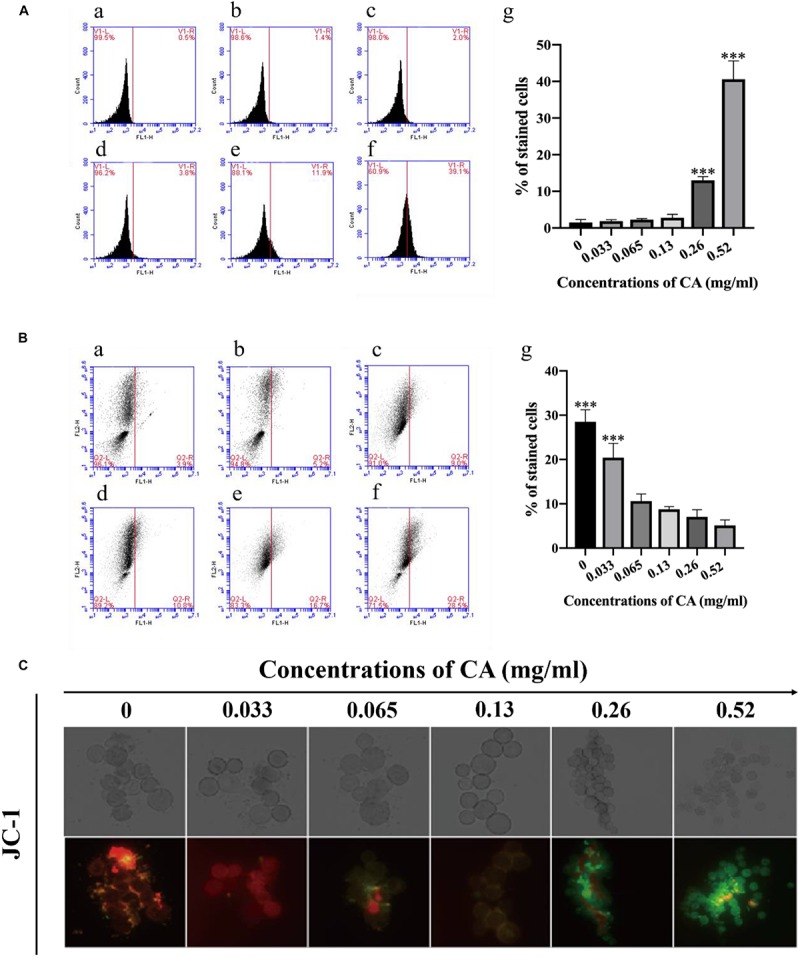
Flow cytometry analysis of ROS content and Δψ*_*m*_* in CA-treated *A. flavus*. **A** **(a)** Fluorescence of cells without CA treatment; **(b–f)** *A. flavus* cells exposed to 0.033, 0.065, 0.13, 0.26, and 0.52 mg/ml CA. **(B)** Statistical analysis of percentage of stained cells. **(C)** CA decreased the extent of mitochondrial damage to cells as detected by flow cytometry and the statistical analysis of the stained cells; **(a)** Fluorescence of cells without CA treatment; **(b–f)** *A. flavus* cells exposed to 0.033, 0.065, 0.13, 0.26, and 0.52 mg/ml CA. **A (g)** and **B (g)** Statistical analysis of percentage of stained cells. **(C)** Fluorescence microscopy analysis of the degree of mitochondrial depolarization. JC-1 generates red fluorescence when the mitochondrial membrane potential is high, and green fluorescence when the mitochondrial membrane potential is low. In all statistical analyses ^∗∗∗^*p* < 0.001 when compared with the 0 mg/ml CA.

In various physiological and pathological processes, ROS plays a vital role in autophagy and in cell death (Xu et al., 2017). We used the sensitive fluorescent dye DCFH-DA to investigate the production of intracellular ROS by flow cytometer. As shown in Figure 4, the generation of intracellular ROS increased significantly after *A. flavus* cells were treated with increasing concentrations of CA. The data indicate that CA may be conducive to an accumulation of intracellular ROS.

### Effect of CA on Δψ*_*m*_* of *A. flavus* Cells

Δψ*_*m*_* is known to promote cell death and to act as a protease in the extracellular matrix (Kimura-Ohba and Yang, 2016). In this study, we used fluorescence microscopy and flow cytometry with JC-1 staining to measure the effect of CA on Δψ*_*m*_* in *A. flavus* cells: a decrease in Δψ*_*m*_* was evident in *A. flavus* cells with increasing concentrations of CA after 12 h of treatment (Figure 4C). As shown in Figure 4, a typical fluorescence distribution of JC-1 was displayed in the non-treated group, and the J-aggregates were red. We found that the cells stained with JC-1 changed to a cytoplasmic formation of J-monomeric (green) forms with increased concentrations of CA. These results indicate that CA may decrease Δψ*_*m*_* in *A. flavus* cells in a concentration-dependent manner.

### CA Increased Cytoplasmic and Mitochondrial Ca^2+^ Levels

**FIGURE 5:**
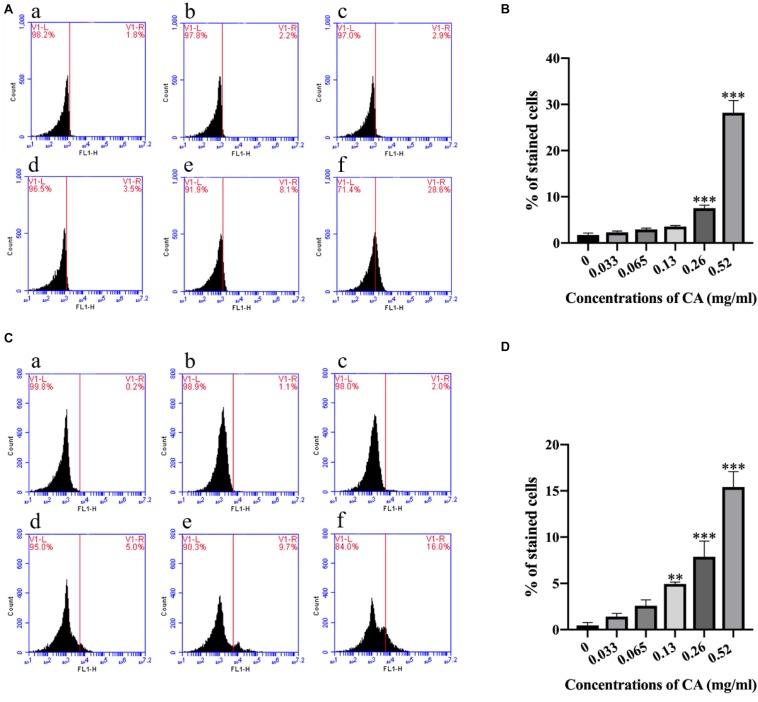
Cinnamaldehyde promoted the Ca^2+^ accumulation in both the cytoplasm and the mitochondria. **(A)** Rhod-2/AM fluorescence probe was used to detect the content of calcium ion in the mitochondria after being treated with different concentrations of CA; **(a)** Fluorescence of cells without CA treatment; **(b–f)** *A. flavus* cells exposed to 0.033, 0.065, 0.13, 0.26, and 0.52 mg/ml CA. **(B)** Statistical analysis of percentage of stained cells. **(C)** Fluo-3/AM fluorescence probe was used to test the concentrations of Ca^2+^ in the cytoplasm after co-incubation with CA; **(a)** Fluorescence of cells without CA treatment; **(b–f)** *A. flavus* cells were treated with 0.033, 0.065, 0.13, 0.26, and 0.52 mg/ml CA. **(D)** Statistical analysis of percentage of stained cells. In all statistical analyses, ^∗∗^*p* < 0.01 and ^∗∗∗^*p* < 0.001 when compared with the 0 mg/ml CA.

Ca^2+^ in the mitochondria plays an important role in the regulation of cell survival, apoptosis, and autophagy (Chemaly et al., 2018), and the Ca^2+^ level in the cytoplasm is always elevated during the process of cell apoptosis (Zhu et al., 2018). In this study, we selected the Fluo-3/AM and Rhod-2/AM stains to detect the levels of Ca^2+^ in the cytoplasm and mitochondria. Compared with the non-treated cells, the Ca^2+^ was increased in the mitochondria at different concentrations of CA (Figures 5A,B). Furthermore, the Ca^2+^ level in the cytoplasm was also elevated with the increasing concentrations of CA (Figures 5C,D). These results suggest that CA may induce an increase in cytoplasmic and mitochondrial Ca^2+^ levels.

### CA Induced the Release of Cytochrome c

**FIGURE 6:**
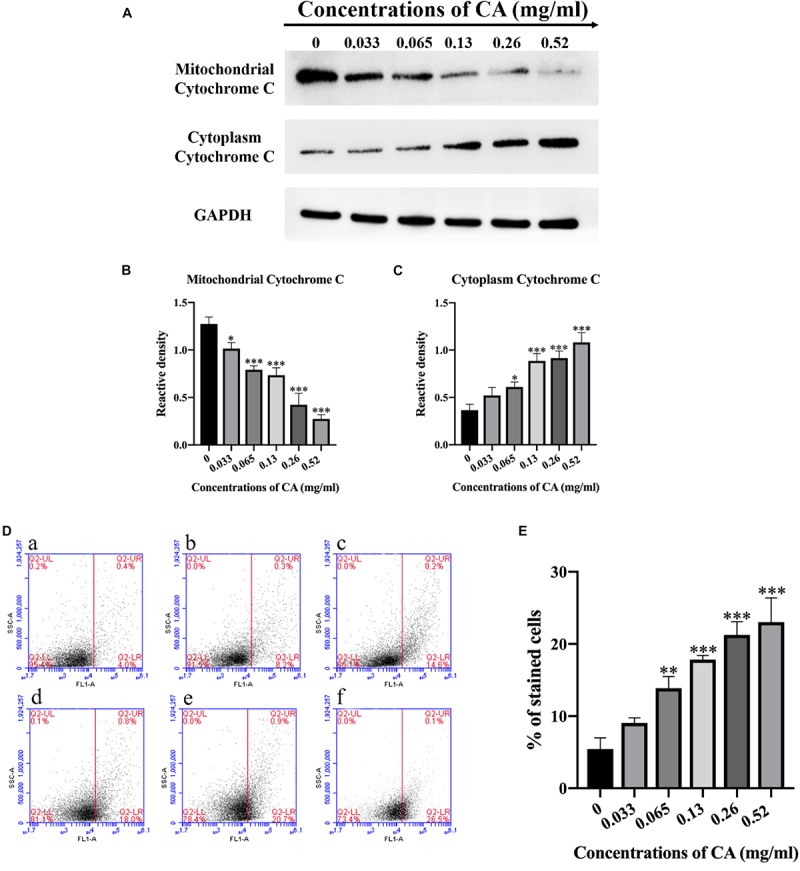
Cinnamaldehyde induced cytochrome c to be released from the mitochondria into the cytoplasm and induced apoptosis of *A. flavus* through the metacaspase pathway. **(A)** Western blot analysis of the content of cytochrome c in mitochondria and cytoplasm. **(B,C)** A gray value analysis of mitochondrial cytochrome c and cytoplasmic cytochrome c. **(D)** **(a)** Fluorescence of cells without CA treatment; **(b–f)** *A. flavus* cells exposed to 0.033, 0.065, 0.13, 0.26, and 0.52 mg/ml CA. **(E)** Statistical analysis of percentage of stained cells. In all statistical analyses, ^∗^*p* < 0.05, ^∗∗^*p* < 0.01, and ^∗∗∗^*p* < 0.001 when compared with the 0 mg/ml CA.

Cytochrome c plays an important role in initiating apoptosis, and its release from the mitochondria is a crucial event in the mammalian cell (Liu et al., 2012). The levels of cytochrome c in the mitochondria and cytoplasm were detected by western blot after *A. flavus* cells were co-incubated with various concentrations of CA. Compared with the non-treated cells, the level of cytochrome c in the mitochondria significantly decreased, while the level in the cytosol increased noticeably (Figures 6A–C). The results demonstrate that CA induced the release of cytochrome c from the mitochondria in *A. flavus* cells.

### CA Caused Activation of Metacaspase

In fungi, plants, and in some bacteria, the metacaspases have been implicated in programmed cell death (PCD) (Asplund-Samuelsson et al., 2012). We stained the cells with CaspACE FITC-VAD-FMK, and the cells were incubated with CA. As shown in Figure 6D, the percentage of *A. flavus* cells that were significantly stained increased in a dose-dependent manner. This result indicates that CA induced the activation of the metacaspases to initiate apoptosis in *A. flavus*.

### CA Caused PS Externalization

**FIGURE 7:**
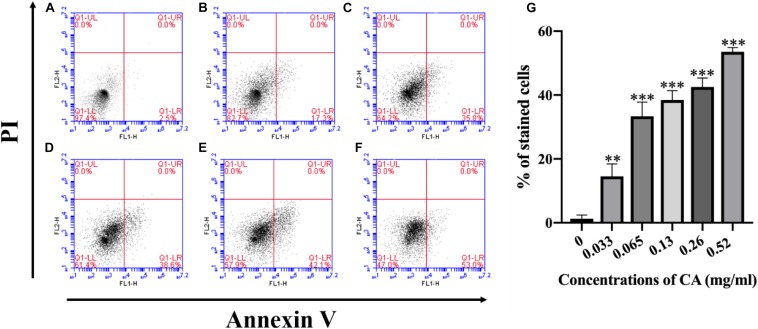
Flow cytometry measures PS externalization in *A. flavus* after being treated with CA. **(A)** Fluorescence of cells without CA treatment. **(B–F)** *A. flavus* cells treated with 0.033, 0.065, 0.13, 0.26, and 0.52 mg/ml CA. **(G)** Statistical analysis of percentage of stained cells. In all statistical analyses, ^∗∗^*p* < 0.01 and ^∗∗∗^*p* < 0.001 when compared with the 0 mg/ml CA.

Phosphatidylserine (PS) is expressed in the outer layers of the cell membrane and is “flipped out” from the inner layers in early apoptosis (Chowdhury et al., 2014). In this assay, *A. flavus* cells were double-stained with Annexin V-FITC and PI at various concentrations of CA treatment, and apoptotic cells were identified by flow cytometry. Figure 7 depicts the percentages of early apoptotic cells (Annexin V-positive and PI-negative) in the lower right quadrant and this increases in a time-concentration-dependent manner. The results conclusively indicate that CA can lead to apoptosis through the externalization of PS.

### Effect of CA on DNA Damage and Nuclear Fragmentation

**FIGURE 8:**
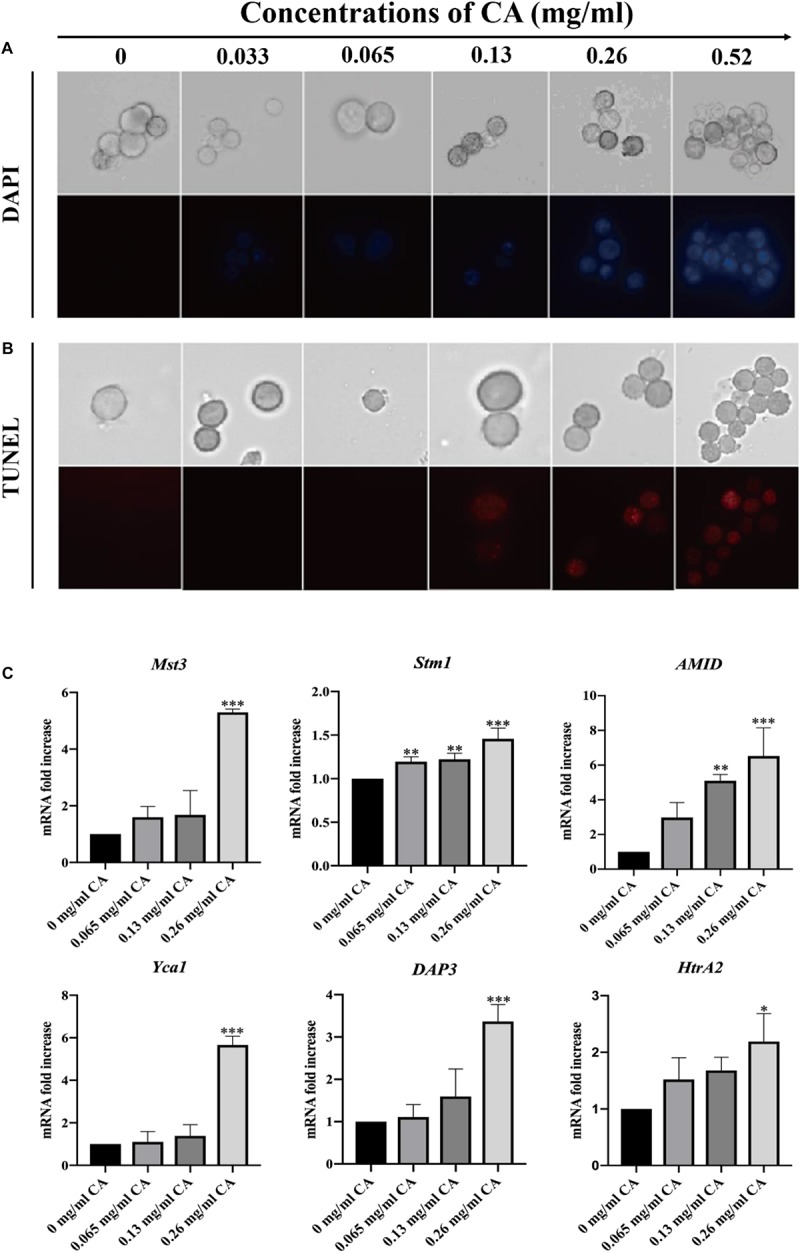
DNA damage and nuclear fragmentation by CA were visualized with fluorescence microscopy using TUNEL and DAPI staining. **(A)** Blue fluorescence indicates a nuclear signal after staining by DAPI. **(B)** Red fluorescence means a positive signal in TUNEL staining. **(C)** The expression levels of apoptosis-related genes (*Mst3*, *Stm1*, *AMID*, *Yca1*, *DAP3*, and *HtrA2*) at various concentrations of CA (0, 0.065, 0.13, and 0.26 mg/ml) were examined by Real-Time PCR. In all statistical analyses, ^∗^*p* < 0.05, ^∗∗^*p* < 0.01, and ^∗∗∗^*p* < 0.001 when compared with the 0 mg/ml CA.

The degree of DNA damage is related to the degree of apoptosis, with DNA damage preceding apoptosis, which is consistent with the time of execution of apoptosis (Rai et al., 2015). We used DAPI and TUNEL staining to detect DNA damage and nuclear fragmentation, which are hallmarks of late apoptosis. In the microscopic analysis, the cells treated for 12 h with various concentrations of CA showed an increasing fluorescence intensity, which indicated CA induced DNA damage (Figure 8B). Similarly, we found that when *A. flavus* cells were exposed to CA they had a DAPI-positive phenotype and showed chromatin condensation, which suggested that the CA induced nuclear fragmentation (Figure 8A). Our results show that CA caused DNA damage and nuclear fragmentation in *A. flavus* cells. In recent years, the literature has demonstrated that *Mst3*, *Stm1*, *AMID*, *Yca1*, *DAP3*, and *HtrA2* are all genes associated with apoptosis (Fedorova et al., 2005). As Figure 8C shows, after 12 h of treatment with CA, the expression levels of *Mst3*, *Stm1*, *AMID*, *Yca1*, *DAP3*, and *HtrA2* were significantly increased. β-tubulin was selected as the reference gene as it displayed the same expression level in different samples. We found that the relative expression levels of the apoptotic genes were significantly changed after *A. flavus* cells were treated with 0.26 mg/ml CA. The results showed that CA can affect the expression of *Mst3*, *Stm1*, *AMID*, *Yca1*, *DAP3*, and *HtrA2*, which then activate related pathways to induce apoptosis in *A. flavus* cells.

## Discussion

Aromatic and medicinal plants have been used as pharmaceutical and food preservatives for decades. Many plants, such as cloves, thyme, and cinnamon, have been used to treat infectious diseases and to protect foods because they have been shown to have antimicrobial activity against spoilage by fungi and bacteria (Liu et al., 2017). Aromatic and medicinal plants produce essential oils in the form of secondary metabolites (Pandey et al., 2016). Essential oils have been reported to have a wide range of antifungal activities (Tian et al., 2011). CA is the main component of the cinnamon essential oil, and this has been developed as a food antimicrobial agent due to its activity against bacteria, yeast, and filamentous mold (Hu et al., 2013). According to the report, during corn storage, a complex essential oil rich in CA reduced the content of aflatoxin B1, zearalenone, and deoxycaprolol. Furthermore, this complex essential oil also showed the capability to reduce the contamination by *Fusarium*, *Wallemia*, *Sarocladium*, and *Penicillium* in the process of maize kernels storage (Wang et al., 2019). CA was reported to be highly safe, 20 times of effective dose (20 mg/kg) of this compound does not cause abnormal behavioral signs and serum chemical damage throughout the study (Subash Babu et al., 2007; Anand et al., 2010). Therefore, the development of CA into a new type of natural preservative has a certain safety and theoretical basis.

This study showed that CA has potential antifungal activity against *A. flavus* and may be a source of natural antifungal compounds that negatively affect the growth of *A. flavus*. The MIC of CA against *A. flavus* is 0.065 mg/ml (Figure 1A). When we applied CA to corn preservation we found that CA inhibited corn spoilage at a concentration of 0.13 mg/cm^3^ (Figure 1B). CA inhibited spore germination and mycelial growth and reduced biomass production (Figure 2). In addition, the morphology of *A. flavus* cells changed after CA treatment (Figures 3A,B). These results are consistent with previous research that reported that *A. flavus* seemed to be shriveled and wrinkled after treatment by CA as shown by scanning electron microscopy (Sun et al., 2016). The ability of CA to inhibit spore germination, mycelium growth, and biomass production in *A. flavus* is consistent with the results of previous studies (Tian et al., 2015; Sun et al., 2016).

Ergosterol is a unique component in the fungal cell membrane and plays a vital role in the activity of fungal cells, where it serves to stabilize the membrane structure, regulate membrane fluidity, and ensure material transportation (de Lira Mota et al., 2012). Most antifungal drugs used clinically target ergosterol or its biosynthesis (Shapiro et al., 2011). When ergosterol synthesis is reduced, the physiological activity of the cell membrane is affected, which is likely to cause fungal cell membrane damage and cell breakage (Georgopapadakou and Walsh, 1994). Some researchers have shown that the lipophilic nature of essential oils allows them to pass easily through the cell membrane to induce biological responses (Tian et al., 2015). We examined the synthesis of ergosterol and detected cell membrane damage by monitoring the uptake of the fluorescent nuclear stain PI. The results showed that when *A. flavus* was treated with different concentrations of CA (0, 0.033, 0.065, 0.13, 0.26, and 0.52 mg/ml) it reduced the synthesis of ergosterol and caused cell membrane damage (Figures 3C–F). This result is consistent with previously reported results that tested products such as citral, octanal, and alpha-terpineol, which can all damage cell membranes with consequent bacteriostatic action (Zhou et al., 2014). We also detected a release of the contents of *A. flavus* and found that CA can cause this release of contents. This result indicated that the cell membrane was damaged (Figure 3G), verifying the previous results.

Apoptosis is a unique form of PCD in which cells activate an intrinsic suicide program for self-destruction (Perez-Garijo et al., 2013). Currently, clinically used antifungal drugs such as peptaibols, anacardic acid, and amphotericin B, which are cytotoxic to pathogenic fungi through activation of an apoptotic pathway (Muzaffar et al., 2016) are considered to offer a promising approach to the prevention of fungi and food contamination. In the light of previous results, we determined that CA can effectively inhibit *A. flavus*. We therefore focused on its mechanism of apoptosis.

The redox state of cells plays a crucial role in cell fate. A slight imbalance between the rate of production and the breakdown of reactive oxygen and nitrogen species (ROS and RNS) may lead to activation of the cell death pathway (Hirpara et al., 2001). It is worth noting that mitochondria are the primary intracellular source of ROS, mainly superoxide (${\text{O}}_{2}^{+}$) and hydrogen peroxide (H_2_O_2_), as electrons are promoted leading to oxygen leakage through high-throughput electron transport chains (ETCs) (Finkel and Holbrook, 2000). The accumulation of ROS is considered to be one of the earliest changes associated with PCD (Tian et al., 2016). With this in mind, a DCFH-DA assay was applied to detect changes in the levels of ROS in CA-treated *A. flavus* cells. Compared with the non-CA-treated *A. flavus* cells, our results indicated that intracellular ROS levels increased significantly in CA-treated *A. flavu*s (Figures 4A,B). The emergence of high levels of ROS can lead to mitochondrial damage, cell membrane damage, and even DNA breaks (Phaniendra et al., 2015). It has also been reported that an increase in Δψ*_*m*_* is associated with high intracellular ROS accumulation (Sukumar et al., 2016).

Mitochondria are essential regulators of cellular bioenergetics, and mitochondria that are damaged by ROS tend to produce more ROS, thereby activating mitochondria-mediated apoptosis or necrosis pathways. The opening of mitochondrial permeability transition pores (MPT pores) leads to a loss of the mitochondrial inner membrane integrity. MPT pores allow flux of small molecules, <1500 Da, and protons, leading to mitochondrial swelling, loss of Δψm, rupture of the outer membrane, and death through apoptosis or necrosis. The formation of these pores can occur in response to several stimuli, including Ca^2+^ overload and oxidative stress (Handy and Loscalzo, 2012). Therefore, we measured changes in Δψm in CA-treated *A. flavus* cells by using a JC-1 probe. As shown in Figure 4C, Δψm significantly decreased after incubation with different concentrations of CA (0, 0.033, 0.065, 0.13, 0.26, and 0.52 mg/ml), and this result is consistent with the results of previous research (Yun et al., 2017). We speculated that the mitochondrial homeostasis is disrupted, causing its dysfunction and leading to changes in membrane potential.

The role of ROS and Ca^2+^ channels may potentially modulate mitochondrial dysfunction, form MPT pores, and induce apoptosis. Ca^2+^ overload can lead to cell death. Calcium, as a major second messenger in cells, is well-known for its important role in mediating PCD (Handy and Loscalzo, 2012). Increased intracellular calcium is a sign of early apoptosis in cells. When the balance of intracellular calcium is disrupted, it leads to the release of cytochrome c and other pro-apoptotic factors (Garcia-Prieto et al., 2013). The results of this study illustrated a concentration-dependent increase in cytoplasmic Ca^2+^ in CA-treated *A. flavus* as well as significant increases in mitochondrial Ca^2+^ levels (Figure 5), which echo the results of previous reports. Overloading of mitochondrial Ca^2+^ disrupts mitochondrial function and depolarization of Δψ*_*m*_*. This result confirms the pathological events leading to apoptosis (Yun and Lee, 2016).

Given the above-mentioned disruption of the intracellular calcium balance leading to the release of cytochrome c, the collapse of Δψ*_*m*_* is closely related to a series of events, including the release of cytochrome c, the activation of metacaspase, DNA damage, and nuclear fragmentation. Cytochrome c is a pro-apoptotic protein, and the opening of the MPT pores causes the mitochondrial membrane rupture to release cytochrome c (Mallick et al., 2015). In our study, we measured the release of cytochrome c from the mitochondria into the cytoplasm in CA-treated *A. flavus* cells by western blot (Figures 6A–C). Release of cytochrome c from mitochondria is a key event initiating apoptosis. It induces the assembly of apoptotic bodies and activates downstream caspase. Further, apoptosis and caspase were initially thought to be crucial markers of apoptosis (Yuan et al., 2016).

In yeast apoptosis, there are caspase-dependent and caspase-independent cell death pathways. The yeast caspase-like protease, known as metacaspase, is encoded by *YCA1*. ROS is a major factor in inducing apoptosis in yeast cells, and it regulates cell death pathways by activating yeast metacaspase (Kim et al., 2016). Therefore, we examined the metacaspase activity of *A. flavus* cells following CA treatment and found that the activity of metacaspase increased in tandem with increased CA concentration, indicating that CA activated metacaspase (Figures 6D,E).

Changes in the phospholipid bilayer in the cell membrane usually occur in the early stages of apoptosis. When apoptosis occurs, the PS component of the phospholipid bilayer will move from the inner membrane to the outer membrane (Tian et al., 2016). We examined the apoptotic characteristics of CA-treated cells, and the results showed that CA caused the externalization of PS on the outer surface of the plasma membrane (Figure 7). DNA damage and nuclear fragmentation are typical morphological features of apoptotic cells in the late stage of apoptosis (Tian et al., 2017). It is well-known that DAPI fluorescent probes are used to detect chromatin condensation, and TUNEL staining is one of the most reliable strategies for identifying the amount of DNA fragmentation visible. Our fluorescence results indicated that CA can significantly affect DNA damage and chromatin condensation in *A. flavus* (Figures 8A,B).

**FIGURE 9:**
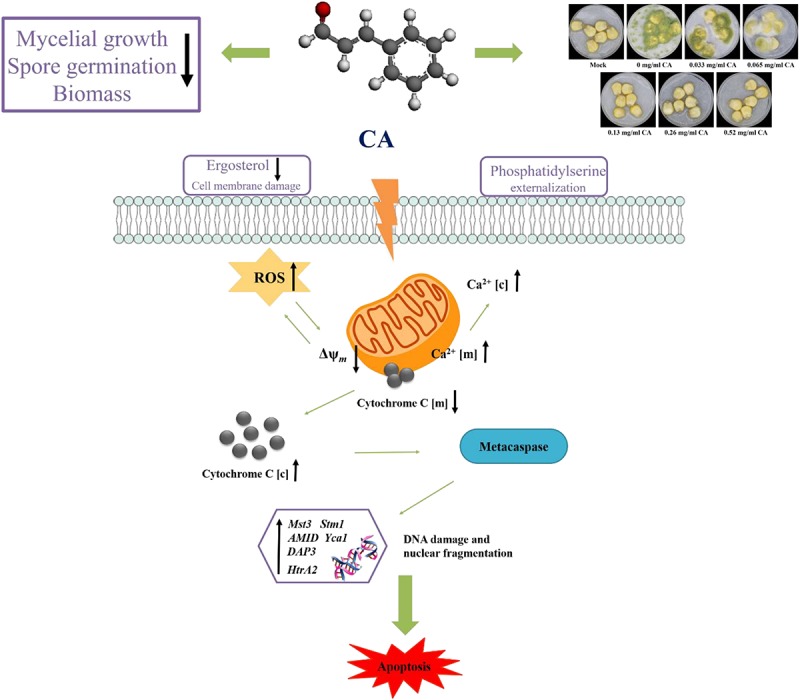
A schematic illustration of the potential inhibition mechanism on *A. flavus* by CA.

In summary, our study demonstrates that Ca^2+^ and ROS-mediated apoptosis can occur in *A. flavus* treated with CA. We propose a model of apoptosis mechanism, as shown in Figure 9. CA causes an increase in Ca^2+^ and ROS. Ca^2+^ overload and oxidative stress disrupt mitochondrial function and cause the loss of Δψ*_*m*_*, which in turn promote the release of cytochrome c from the mitochondria into the cytoplasm. PS externalization can be observed in the early stages of apoptosis, and the increase in ROS activates metacaspase, which further induces apoptosis. Finally, typical morphological features of late apoptosis, DNA fragmentation, and chromatin condensation can be observed.

To further explore the molecular mechanism of CA-induced apoptosis in *A. flavus*, we examined apoptosis-related genes by RT-PCR. As shown in Figure 8C, the expression levels of *Mst3*, *Stm1*, *AMID*, and *Yca1* increased in tandem with an increase in CA concentration. Previous studies have shown that these genes are all coding for the caspase family in fungi (Fedorova et al., 2005), which is consistent with our finding that CA activates metacaspase. Over-expression of these genes may be a potential mechanism by which CA activates metacaspase to induce apoptosis. The expression levels of *DAP3* and *HtrA2* exhibited the same trend, showing concentration-dependence. These two genes have been reported to be involved in mitochondrial homeostasis and injury (Fedorova et al., 2005). Over-expression of these two genes may cause CA to disrupt mitochondrial homeostasis, lead to mitochondrial damage, promote the release of apoptotic factors, and ultimately result in apoptosis.

## Conclusion

Cinnamaldehyde can inhibit mycelial growth, buccal germination, and biomass production. It can alter cell morphology, cause cell membrane damage, and cause mitochondrial dysfunction through the interaction between Ca^2+^ and ROS, leading to apoptosis of *A. flavus*. CA is also effective in preventing corn spoilage. The results of this study indicate that CA is a potential candidate for use as a antifungal agent in food preservation.

## Data Availability Statement

The raw data supporting the conclusions of this article will be made available by the authors, without undue reservation, to any qualified researcher.

## Author Contributions

JT, KY, and YGL designed the experiments. SQ, LC, QG, and XH performed the experiments. KY, ML, and YXL analyzed the data. SQ and LC drafted the manuscript. All authors read and approved the final manuscript.

## Conflict of Interest

The authors declare that the research was conducted in the absence of any commercial or financial relationships that could be construed as a potential conflict of interest.
